# Serum Anion Gap Predicts All-Cause Mortality in Critically Ill Patients with Acute Kidney Injury: Analysis of the MIMIC-III Database

**DOI:** 10.1155/2020/6501272

**Published:** 2020-01-19

**Authors:** Bihuan Cheng, Diwen Li, Yuqiang Gong, Binyu Ying, Benji Wang

**Affiliations:** Department of Anesthesiology, Critical Care and Pain Medicine, The Second Affiliated Hospital and Yuying Children's Hospital of Wenzhou Medical University, Wenzhou, 325000 Zhejiang, China

## Abstract

**Background:**

No epidemiological study has investigated the effect of anion gap (AG) on the prognosis of critically ill patients with acute kidney injury (AKI). Therefore, we aimed to determine the association between serum AG and all-cause mortality in these patients.

**Methods:**

From MIMIC III, we extracted demographics, vital signs, laboratory tests, comorbidities, and scoring systems from the first 24 h after patient ICU admission. A generalized additive model was used to identify a nonlinear association between anion gap and 30-day all-cause mortality. We also used the Cox proportional hazards models to measure the association between AG levels and 30-day, 90-day, and 365-day mortality in patients with AKI.

**Results:**

A total of 11,573 eligible subjects were extracted from the MIMIC-III. The relationship between AG levels and 30-day all-cause mortality in patients with AKI was nonlinear, with a U-shaped curve. In multivariate analysis, after adjusting for potential confounders, higher AG was a significant predictor of 30-day, 90-day, and 365-day all-cause mortality compared with lower AG (HR, 95% CI: 1.54, 1.33–1.75; 1.55, 1.38–1.73; 1.46, 1.31–1.60).

**Conclusions:**

The relationship between AG levels and 30-day all-cause mortality described a U-shaped curve. High-AG levels were associated with increased risk 30-day, 90-day, and 365-day all-cause mortality in critically ill patients with AKI.

## 1. Introduction

Acute kidney injury (AKI) is a common syndrome characterized by an abrupt, usually reversible decline in glomerular filtration, associated with substantial morbidity and mortality, especially in critically ill patients [1, 2]. In the US, approximately 20% of critically ill patients have AKI in the intensive care unit (ICU) [3]. In the presence of AKI, patient mortality within 1 year after ICU admission is significantly elevated, as high as 60%–70% [4, 5]. Because of the high incidence and poor prognosis of AKI in critically ill patients, researchers are attempting to identify prognostic predictors in AKI [6, 7]. Unfortunately, most of them are not widely used in clinical practice.

The serum anion gap (AG) is a mathematical derivation parameter calculated from the difference in serum cation and anion concentrations. It is the simplest way to assess acid-base status [8, 9] and helps to identify various forms of metabolic acidosis. AG is also one of the most commonly used biomarkers that provides important clues regarding diagnosis or prognosis of various disorders [10–12]. There is a positive monotonic relationship between high AG and severity of illness or poor prognosis for sepsis [13], coronary artery disease (CAD) [14], aortic aneurysm [15], and chronic kidney disease (CKD) [16]. To the best of our knowledge, no epidemiological study has investigated the effect of AG on the prognosis of critically ill patients with AKI. Therefore, we aimed to determine the association between serum AG and all-cause mortality in these patients.

## 2. Methods

### 2.1. Data Source

We followed the methods of Wang et al. in this study, as we have done previously [17–19]. Our study is based on an openly available clinical database called the Multiparameter Intelligent Monitoring in Intensive Care III (MIMIC III) [20]. The database includes more than 40,000 ICU patients admitted to Beth Israel Deaconess Medical Center (Boston, MA, USA) from 2001 to 2012. To apply for access to the database, we completed the National Institutes of Health's web-based course and passed the Protecting Human Research Participants exam (no. 6182750). We extracted clinical variables, including demographic characteristics, International Classification of Diseases (ICD-9) codes, physiological index, medications, and laboratory tests. The project was approved by the Institutional Review Boards of Beth Israel Deaconess Medical Center and the Massachusetts Institute of Technology (Cambridge, MA). To safeguard patient privacy, data were deidentified; therefore, informed consent was waived.

### 2.2. Population Selection Criteria

Adult patients (≥18 years) with AKI according to ICD-9 code at first ICU admission for more than two days were included. The exclusion criteria were (1) missing AG at ICU admission and (2) missing >5% individual data.

### 2.3. Data Extraction

Structured Query Language (SQL) with PostgreSQL (version 9.6) was used to extract data from MIMIC-III. Demographics, vital signs, laboratory tests, comorbidities, scoring systems, and other variables collected within the first 24 h of ICU admission were extracted from MIMIC III. The comorbidities included CAD, congestive heart failure (CHF), atrial fibrillation (AFIB), stroke, renal disease, liver disease, pneumonia, respiratory failure, and acute respiratory distress syndrome (ARDS). Laboratory data were also extracted, including AG, albumin, bicarbonate, bilirubin, creatinine, chloride, glucose, hematocrit, hemoglobin, platelet, sodium, potassium, lactate, blood urea nitrogen (BUN), white blood cell (WBC), prothrombin time (PT), activated partial thromboplastin time (APTT), and international normalized ratio (INR). We also calculated the sequential organ failure assessment (SOFA) score [21] and simplified acute physiology score II (SAPSII) [22] for each patient. Other extracted data included age, gender, systolic blood pressure (SBP), diastolic blood pressure (DBP), mean blood pressure (MBP), heart rate, respiratory rate, temperature, SPO2, AKI stage, renal replacement therapy, and ICU length of stay (LOS). Survival information on vital status was obtained from Social Security Death Index records. The endpoints of our study were 30-day, 90-day, and 365-day all-cause mortality from the date of ICU admission.

### 2.4. Statistical Analysis

Continuous variables were expressed as mean ± SD or medians and interquartile range (IQR). Categorical data were expressed as frequencies or percentages. Chi-square, 1-way ANOVA, and Kruskal-Wallis *H* tests were used to determine any significant differences between the groups. A generalized additive model (GAM) was used to identify the nonlinear association between AG and 30-day all-cause mortality. We also used the Cox proportional hazards models to determine associations between AG levels and 30-day, 90-day, and 365-day mortality in AKI; these results were expressed as hazard ratios (HRs) with 95% confidence intervals (CIs).

Variables based on epidemiological and biological background were incorporated as potential confounders, and those confounders based on a change in effect estimate of >10% were used to generate an adjusted model [23]. For each endpoint, two multivariate models were constructed on the basis of AG group inclusion according to tertiles. The first tertile was treated as the reference group. In model I, covariates were adjusted for age and gender. In model II, we further adjusted for age, gender, AKI stage, CHF, CAD, liver disease, stroke, respiratory failure, pneumonia, SIRS, potassium, albumin, lactate, platelet, BUN, PT, INR, APTT, WBC, pH, creatinine, bicarbonate, bilirubin, renal replacement therapy, respiration rate, SPO2, heart rate, SBP, DBP, temperature, Elixhauser comorbidity index, SOFA, and SAPSII. Subgroup analysis of the associations between AG and 30-day all-cause mortality was performed using stratified linear regression models. All probability values were 2-sided, and values less than 0.05 were considered statistically significant. R (http://www.R-project.org) and EmpowerStats (http://www.empowerstats.com/en/, X&Y solutions, Inc., Boston, MA) were used for all statistical analysis.

## 3. Results

### 3.1. Subject Characteristics

The patients were divided according to AG in tertiles, and baseline characteristics of these patients are summarized in Table 1. A total of 11,573 eligible subjects were extracted from the MIMIC-III. There were 6,626 men and 4,947 women, and patients were generally older. Patients with higher AG (AG ≥ 14) were more likely to report a history of CAD, CHF, AFIB, and renal disease and had higher values of bilirubin, creatinine, potassium, lactate, BUN, WBC, PT, and APTT. SAPSII, SOFA scores, mortality, use of RRT, and ICU LOS were also significantly higher in the high-AG group (AG ≥ 14) than in the lower-AG group (AG< 12).

### 3.2. AG Levels and All-Cause Mortality

**Figure 1:**
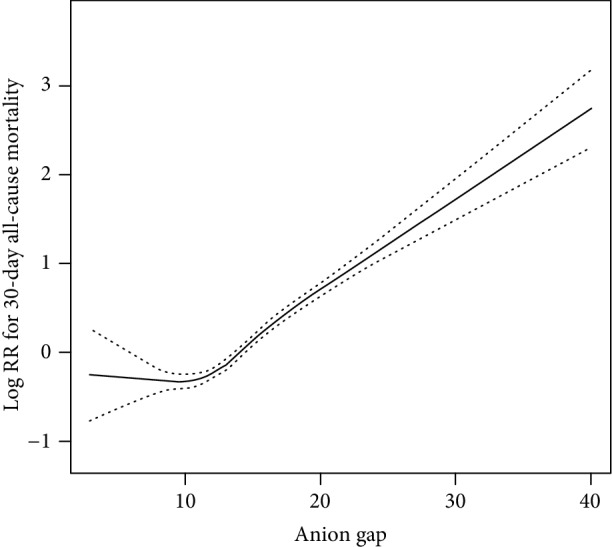
Association between anion gap and 30-day all-cause mortality. A threshold, nonlinear association between anion gap and 30-day all-cause mortality was found in a generalized additive model (GAM). Solid rad line represents the smooth curve fit between variables. Imaginary lines represent the 95% of confidence interval from the fit.

The relationship between AG levels and 30-day all-cause mortality was nonlinear, and a U-shaped curve was observed (Figure 1). We used Cox proportional hazards regression model to determine the associations between AG and 30-day, 90-day, and 365-day all-cause mortality in patients with AKI (Table 2). In model I, high-AG levels (AG ≥ 14) were associated with increased risk of all-cause mortality after adjustment for age and gender. In model II, the lower AG (AG < 12) was used as a reference. After adjustment for confounders (age, gender, acute kidney injury stage, congestive heart failure, coronary artery disease, liver disease, stroke, respiratory failure, pneumonia, SIRS, potassium, albumin, lactate, platelet, BUN, PT, INR, APTT, WBC, pH, creatinine, bicarbonate, sodium, chloride, diabetes, bilirubin, renal replacement therapy, respiration rate, SPO2, heart rate, SBP, DBP, temperature, Elixhauser comorbidity index, SOFA, and SAPSII), higher AG (AG ≥ 14) remained a more significant predictor of 30-day, 90-day, and 365-day all-cause mortality than lower AG (HR, 95% CI: 1.54, 1.33–1.75; 1.55, 1.38–1.73; 1.46, 1.31–1.60). For the purpose of sensitivity analysis, we also handled AG as categorical variable (tertile) and found the same trend (*P* for trend: <0.0001). In addition, in order to verify that AG was an independent prognostic factor for AKI, we also analyzed the potential associations of bicarbonate, pH, lactate, and urine ketone bodies on all-cause mortality, and the results were included in supplementary material ([Supplementary-material supplementary-material-1]).

### 3.3. Subgroup Analyses

As shown in Table 3, the test for interactions was statistically significant in several strata (*P* for interaction <0.05). Among these strata, we observed that patients with higher AGs had significantly higher mortality with hypotension, bicarbonate < 24 mg/dL, bilirubin ≥ 0.7 mg/dL, lactate ≥ 2.5 mmol/L, PT ≥ 15.2 s, INR ≥ 1.4, APTT ≥ 34.8 s, WBC ≥ 13 × 10^9^/L, creatinine ≥ 1.4 mEq/L, and chloride ≥ 107 mmol/L. Similar trends were observed in patients with CAD and liver disease.

## 4. Discussion

The relationship between AG and 30-day all-cause mortality among critically ill patients with AKI was nonlinear, and a U-shaped curve was observed. In the fully adjusted model, high-AG levels were associated with increased risk 30-day, 90-day, and 365-day all-cause mortality. To our knowledge, this was the first study to measure the association between serum AG and all-cause mortality in critically ill patients with AKI.

Several studies have explored the relationship between AG and clinical outcomes of various diseases. Yang et al. [14] measured the association between the AG and all-cause mortality in CAD and found that higher AG was associated with worse cardiac function and was a significant predictor of all-cause mortality. Banerjee et al. [24] suggested that greater AG was present among persons with CKD, and AG increased the risk for progression to end-stage renal disease in these patients. AG is a traditional tool for assessing acid-base status, and most previous studies have associated it with acid-base disorders, all of which have significant impacts on morbidity and mortality in critically ill patients [25]. Similarly, our findings showed a positive correlation between serum AG and all-cause mortality in critically ill patients with AKI. Nevertheless, the underlying mechanism requires further research.

AG reflects the unmeasured anion concentration and can be easily calculated from conventional clinical chemical analysis. It is widely used to evaluate the acid-base status and is one of the most commonly used biomarkers that provides important clues for the diagnosis and prognosis of various diseases [13, 15]. Elevated serum AG is usually caused by overproduction of organic acid anions and/or reduction in anion excretion [8]; increased serum lactate and ketoanions are the main reasons for increased AG [26]. AKI is defined as rapid decline in renal function lasting from hours to days, as opposed to chronic kidney disease [27]. Critically ill patients are often exposed to hypoxia and anaerobic tissue conditions, leading to rapid accumulation of pyruvate, which is almost completely converted to lactate [28]. The kidney is known for lactate dehydrogenase dysfunction in the setting of patients with AKI [29], and the renal acid excretion does not fully offset endogenous acid production [30]. Consequently, high AG is common in critically ill patients with AKI.

Similar statistical methods were used to analyze the relationship between lactate, bicarbonate, pH, and urine ketone bodies on the prognosis of AKI patients, as shown in supplementary material. In the fully adjusted model, lactate levels were associated with increased risk 30-day and 90-day all-cause mortality among critically ill patients with AKI, but not with 365-day mortality. As we all know, lactate is closely related to the prognosis of critically ill patients, especially the risk of short-term death [31, 32], which can explain the correlation between lactate and short-term prognosis of critically ill patients with AKI. Moreover, bicarbonate, pH, and urine ketone bodies were not independently associated with 30-day, 90-day, and 365-day all-cause mortality. Interestingly, our results further suggested a positive correlation between AG and all-cause mortality in these patients after adjusting for potential confounders such as lactate, pH, and bicarbonate.

There are several limitations in the present study. First, this was a single-center retrospective observational study, and selection bias was inevitable. Second, we measured serum AG in patients only upon admission to the ICU and did not have laboratory follow-up data. There is the possibility of misclassified measured data that may influence the summary results. Third, although we adjusted for confounding factors, our results may have been influenced by other unknown factors. Finally, we could not determine the underlying mechanism between higher AG and poor prognosis; therefore, further study regarding the mechanism is necessary.

## 5. Conclusions

We found that the relationship between AG levels and 30-day all-cause mortality was nonlinear, with a U-shaped curve. High-AG levels were associated with increased risk 30-day, 90-day, and 365-day all-cause mortality in critically ill patients with AKI.

## Figures and Tables

**Table 1:** Baseline characteristics of participants according to anion gap (*N* = 11,573).

Characteristic	Anion gap (mmol/L)			*P* value
	Q1 (<12)	Q2 (≥12-<14)	Q3 (≥14)	
Age (years)	62.4 ± 17.3	64.5 ± 16.9	64.9 ± 16.6	<0.001
Gender, *n* (%)				0.653
Female	1432 (42.2)	1312 (43.3)	2203 (42.8)	
Male	1964 (57.8)	1718 (56.7)	2944 (57.2)	
MBP (mmHg)	77.5 ± 11.2	77.8 ± 11.5	77.7 ± 12.8	0.316
Heart rate (beats/minute)	87.9 ± 16.9	87.5 ± 16.8	89.0 ± 17.3	<0.001
Respiratory rate (beats/minute)	18.9 ± 4.1	19.4 ± 4.2	20.1 ± 4.4	<0.001
Temperature (°C)	36.9 ± 0.7	36.9 ± 0.7	36.8 ± 0.7	<0.001
SPO2 (%)	97.4 ± 2.0	97.3 ± 2.2	97.0 ± 2.8	<0.001
Comorbidities, *n* (%)				
Coronary artery disease	719 (21.2)	709 (23.4)	1224 (23.8)	0.015
Congestive heart failure	503 (14.8)	540 (17.8)	1131 (22.0)	<0.001
Atrial fibrillation	774 (22.8)	858 (28.3)	1546 (30.0)	<0.001
Stroke	224 (6.6)	275 (9.1)	408 (7.9)	0.001
Renal disease	387 (11.4)	479 (15.8)	1381 (26.8)	<0.001
Liver disease	470 (13.8)	315 (10.4)	605 (11.8)	<0.001
Respiratory failure	1406 (41.4)	1212 (40.0)	2141 (41.6)	0.339
ARDS	62 (1.8)	75 (2.5)	135 (2.6)	0.051
Laboratory parameters				
Anion gap (mmol/L)	9.8 ± 1.4	12.5 ± 0.5	16.5 ± 2.9	<0.001
Albumin (g/dL)	3.1 ± 0.7	3.2 ± 0.7	3.2 ± 0.7	<0.001
Bicarbonate (mmol/L)	26.8 ± 5.1	24.8 ± 4.1	22.6 ± 4.5	<0.001
Bilirubin (mg/dL)	1.8 ± 3.5	1.7 ± 3.9	2.6 ± 6.0	<0.001
Creatinine (mEq/L)	1.3 ± 0.8	1.7 ± 1.2	3.0 ± 2.8	<0.001
Chloride (mmol/L)	109.2 ± 7.5	108.2 ± 6.9	105.9 ± 6.9	<0.001
Glucose (mg/dL)	202.1 ± 150.4	201.4 ± 134.7	203.7 ± 130.3	0.005
Hematocrit (%)	35.5 ± 6.2	35.7 ± 6.0	35.2 ± 6.1	<0.001
Hemoglobin (g/dL)	11.8 ± 2.1	11.9 ± 2.1	11.7 ± 2.1	<0.001
Platelet (10^9^/L)	236.2 ± 129.5	253.8 ± 143.3	251.6 ± 137.7	<0.001
Sodium (mmol/L)	141.0 ± 6.0	140.7 ± 5.3	140.1 ± 5.4	<0.001
Potassium (mmol/L)	4.7 ± 0.9	4.7 ± 1.0	5.0 ± 1.1	<0.001
Lactate (mmol/L)	3.0 ± 2.5	3.3 ± 2.7	3.9 ± 3.4	<0.001
BUN (mg/dL)	27.6 ± 17.6	32.5 ± 20.8	49.1 ± 33.3	<0.001
WBC (10^9^/L)	14.0 ± 12.8	14.8 ± 13.4	16.0 ± 12.9	<0.001
PT (seconds)	17.6 ± 9.1	17.9 ± 11.4	19.8 ± 14.0	<0.001
APTT (seconds)	46.9 ± 32.3	46.2 ± 32.1	49.8 ± 34.3	<0.001
Scoring systems				
SOFA	5.1 ± 3.1	5.1 ± 3.3	6.4 ± 3.8	<0.001
SAPSII	37.7 ± 13.6	38.9 ± 14.0	44.0 ± 15.3	<0.001
AKI stage, *n* (%)				<0.001
Stage 1	838 (24.7)	727 (24.0)	1011 (19.6)	
Stage 2	705 (20.8)	550 (18.2)	689 (13.4)	
Stage 3	1853 (54.6)	1753 (57.9)	3447 (67.0)	
Renal replacement therapy, *n* (%)	154 (4.5)	209 (6.9)	1120 (21.8)	<0.001
ICU LOS (days)	5.9 ± 7.0	6.1 ± 7.2	6.6 ± 8.4	<0.001
30-day mortality, *n* (%)	516 (15.2)	512 (16.9)	1361 (26.4)	<0.001
90-day mortality, *n* (%)	737 (21.7)	729 (24.1)	1796 (34.9)	<0.001
365-day mortality, *n* (%)	1058 (31.2)	1057 (34.9)	2305 (44.8)	<0.001

MBP: mean blood pressure; ARDS: acute respiratory distress syndrome; BUN: blood urea nitrogen; WBC: white blood cell; PT: prothrombin time; APTT: activated partial thromboplastin time; SOFA: sequential organ failure assessment; SAPSII: simplified acute physiology score II; AKI: acute kidney injury; ICU: intensive care unit; LOS: length of stay.

**Table 2:** Relationship between anion gap and all-cause mortality in different models.

Variable	Crude model		Model I		Model II	
	HR (95% CIs)	*P* value	HR (95% CIs)	*P* value	HR (95%CIs)	*P* value
30-day all-cause mortality						
Anion gap (mmol/L)	1.11 (1.10, 1.12)	<0.0001	1.11 (1.10, 1.12)	<0.0001	1.07 (1.06, 1.09)	<0.0001
Anion gap (tertile) (mmol/L)						
<12	1.0 (ref)		1.0 (ref)		1.0 (ref)	
≥12, <14	1.13 (1.00, 1.27)	0.0568	1.08 (0.96, 1.22)	0.2017	1.08 (0.91, 1.26)	0.3774
≥14	1.89 (1.71, 2.09)	<0.0001	1.81 (1.63, 2.00)	<0.0001	1.54 (1.33, 1.75)	<0.0001
*P* for trend	<0.0001		<0.0001		<0.0001	
90-day all-cause mortality						
Anion gap (mmol/L)	1.10 (1.09, 1.11)	<0.0001	1.10 (1.09, 1.11)	<0.0001	1.08 (1.06, 1.10)	<0.0001
Anion gap (tertile) (mmol/L)						
<12	1.0 (ref)		1.0 (ref)		1.0 (ref)	
≥12, <14	1.13 (1.02, 1.25)	0.0216	1.08 (0.98, 1.20)	0.1340	1.09 (0.95, 1.25)	0.1452
≥14	1.78 (1.64, 1.94)	<0.0001	1.70 (1.56, 1.85)	<0.0001	1.55 (1.38, 1.73)	<0.0001
*P* for trend	<0.0001		<0.0001		<0.0001	
365-day all-cause mortality						
Anion gap (mmol/L)	1.08 (1.08, 1.09)	<0.0001	1.09 (1.08, 1.09)	<0.0001	1.08 (1.06, 1.09)	0.0002
Anion gap (tertile) (mmol/L)						
<12	1.0 (ref)		1.0 (ref)		1.0 (ref)	
≥12, <14	1.14 (1.05, 1.25)	0.0019	1.10 (1.01, 1.19)	0.0344	1.14 (1.03, 1.24)	0.0122
≥14	1.64 (1.52, 1.76)	<0.0001	1.56 (1.45, 1.68)	<0.0001	1.46 (1.31, 1.60)	<0.0001
*P* for trend	<0.0001		<0.0001		<0.0001	

HR: hazard ratio; CI: confidence interval. Models were derived from Cox proportional hazards regression models. Crude model adjusted for: none. Model I adjusted for: age and gender. Model II adjusted for: age, gender, acute kidney injury stage, congestive heart failure, coronary artery disease, liver disease, stroke, respiratory failure, pneumonia, SIRS, potassium, albumin, platelet, BUN, PT, INR, APTT, WBC, creatinine, lactate, pH, bicarbonate, sodium, chloride, diabetes, bilirubin, renal replacement therapy, respiration rate, SPO2, heart rate, systolic blood pressure, diastolic blood pressure, temperature, Elixhauser comorbidity index, SOFA, and SAPSII.

**Table 3:** Subgroup analysis of the associations between anion gap and 30-day all-cause mortality.

Characteristic	*N*	HR (95% CI)	*P* value	*P* for interaction
CHF				0.0848
No	9399	1.12 (1.11, 1.13)	<0.0001	
Yes	2174	1.09 (1.06, 1.12)	<0.0001	
CAD				0.0056
No	8921	1.10 (1.09, 1.12)	<0.0001	
Yes	2652	1.15 (1.12, 1.18)	<0.0001	
AFIB				0.0023
No	8395	1.12 (1.11, 1.14)	<0.0001	
Yes	3178	1.09 (1.07, 1.11)	<0.0001	
Renal disease				0.4628
No	9326	1.12 (1.11, 1.13)	<0.0001	
Yes	2247	1.11 (1.09, 1.14)	<0.0001	
Liver disease				0.0436
No	10183	1.11 (1.09, 1.12)	<0.0001	
Yes	1390	1.14 (1.11, 1.16)	<0.0001	
Stroke				0.0184
No	10666	1.12 (1.11, 1.13)	<0.0001	
Yes	907	1.07 (1.03, 1.11)	0.0002	
Pneumonia				<0.0001
No	7997	1.14 (1.12, 1.15)	<0.0001	
Yes	3576	1.06 (1.05, 1.08)	<0.0001	
Respiratory failure				0.0002
No	6814	1.13 (1.12, 1.15)	<0.0001	
Yes	4759	1.09 (1.08, 1.11)	<0.0001	
ARDS				0.8864
No	11301	1.11 (1.10, 1.12)	<0.0001	
Yes	272	1.13 (1.06, 1.19)	<0.0001	
AKI stage				0.0005
Stage 1	2576	1.13 (1.10, 1.16)	<0.0001	
Stage 2	1944	1.05 (1.02, 1.09)	0.0032	
Stage 3	7053	1.11 (1.10, 1.13)	<0.0001	
Albumin (g/dL)				0.3737
<3.2	5614	1.11 (1.10, 1.13)	<0.0001	
≥3.2	5959	1.12 (1.10, 1.14)	<0.0001	
Bicarbonate (mmol/L)				0.0004
<24	5009	1.12 (1.10, 1.13)	<0.0001	
≥24	6564	1.07 (1.05, 1.09)	<0.0001	
Bilirubin (mg/dL)				<0.0001
<0.7	4674	1.07 (1.05, 1.09)	<0.0001	
≥0.7	5521	1.13 (1.12, 1.14)	<0.0001	
Sodium (mmol/L)				0.0608
<140	4737	1.12 (1.10, 1.13)	<0.0001	
≥140	6836	1.11 (1.09, 1.12)	<0.0001	
Potassium (mmol/L)				0.4309
<4.6	5580	1.10 (1.09, 1.12)	<0.0001	
≥4.6	5993	1.11 (1.10, 1.12)	<0.0001	
Lactate (mmol/L)				<0.0001
<2.5	4334	1.06 (1.03, 1.08)	<0.0001	
≥2.5	4416	1.12 (1.11, 1.14)	<0.0001	
BUN (mg/dL)				0.0610
<30	5689	1.12 (1.10, 1.15)	<0.0001	
≥30	5884	1.09 (1.08, 1.10)	<0.0001	
PT (seconds)				<0.0001
<15.2	5464	1.07 (1.05, 1.09)	<0.0001	
≥15.2	5602	1.12 (1.11, 1.13)	<0.0001	
INR				<0.0001
<1.4	4976	1.07 (1.04, 1.09)	<0.0001	
≥1.4	6088	1.11 (1.10, 1.13)	<0.0001	
APTT (seconds)				<0.0001
<34.8	5501	1.07 (1.05, 1.09)	<0.0001	
≥34.8	5544	1.12 (1.11, 1.13)	<0.0001	
WBC (10^9^/L)				0.0245
<13	5755	1.10 (1.09, 1.12)	<0.0001	
≥13	5808	1.11 (1.10, 1.12)	<0.0001	
Platelet (10^9^/L)				0.0888
<226	5758	1.12 (1.11, 1.13)	<0.0001	
≥226	5805	1.10 (1.08, 1.12)	<0.0001	
Hematocrit (%)				<0.0001
<34.9	5770	1.08 (1.07, 1.10)	<0.0001	
≥34.9	5796	1.15 (1.13, 1.17)	<0.0001	
Hemoglobin (g/dL)				<0.0001
<11.6	5699	1.09 (1.07, 1.10)	<0.0001	
≥11.6	5860	1.14 (1.13, 1.16)	<0.0001	
Creatinine (mEq/L)				<0.0001
<1.4	5416	1.06 (1.04, 1.09)	<0.0001	
≥1.4	6157	1.11 (1.10, 1.13)	<0.0001	
Glucose (mg/dL)				0.3993
<165	5744	1.11 (1.09, 1.13)	<0.0001	
≥165	5828	1.11 (1.10, 1.13)	<0.0001	
Chloride (mmol/L)				0.0067
<107	5118	1.10 (1.09, 1.12)	<0.0001	
≥107	6455	1.13 (1.11, 1.15)	<0.0001	
SBP (mmHg)				<0.0001
<114	5763	1.13 (1.11, 1.14)	<0.0001	
≥114	5777	1.08 (1.06, 1.09)	<0.0001	
DBP (mmHg)				0.0099
<60	5761	1.12 (1.11, 1.14)	<0.0001	
≥60	5779	1.09 (1.08, 1.11)	<0.0001	
MBP (mmHg)				0.0004
<76	5765	1.12 (1.11, 1.14)	<0.0001	
≥76	5782	1.09 (1.07, 1.11)	<0.0001	
Heart rate (beats/minute)				0.5626
<87	5767	1.11 (1.09, 1.13)	<0.0001	
≥87	5781	1.11 (1.10, 1.13)	<0.0001	
Respiratory rate (beats/minute)				0.4296
<19	5760	1.10 (1.08, 1.12)	<0.0001	
≥19	5777	1.11 (1.09, 1.12)	<0.0001	
Temperature (°C)				0.0080
<36.8	5717	1.10 (1.09, 1.11)	<0.0001	
≥36.8	5725	1.12 (1.10, 1.14)	<0.0001	
SPO2 (%)				0.3747
<97	5779	1.12 (1.10, 1.13)	<0.0001	
≥97	5765	1.11 (1.09, 1.12)	<0.0001	
SOFA score				0.0513
<5	4872	1.08 (1.05, 1.11)	<0.0001	
≥5	6701	1.09 (1.08, 1.10)	<0.0001	
SAPSII score				0.0306
<39	5479	1.08 (1.05, 1.11)	<0.0001	
≥39	6094	1.08 (1.07, 1.09)	<0.0001	
RRT				<0.0001
No	10090	1.12 (1.11, 1.14)	<0.0001	
Yes	1483	1.06 (1.04, 1.08)	<0.0001	

CHF: congestive heart failure; CAD: coronary artery disease; AFIB: atrial fibrillation; ARDS: acute respiratory distress syndrome; AKI: acute kidney injury; BUN: blood urea nitrogen; PT: prothrombin time; INR: international normalized ratio; APTT: activated partial thromboplastin time; WBC: white blood cell; SBP: systolic blood pressure; DBP: diastolic blood pressure; MBP: mean blood pressure; SOFA: sequential organ failure assessment; SAPSII: simplified acute physiology score II; RRT: renal replacement therapy.

## Data Availability

The clinical data used to support the findings of this study were supplied by Monitoring in Intensive Care Database III version 1.4 (MIMIC-III v.1.4). Although the database is publicly and freely available, researchers must complete the National Institutes of Health's web-based course known as Protecting Human Research Participants to apply for permission to access the database.

## Supplementary Materials

Supplementary Materials Table 1: relationship between bicarbonate and all-cause mortality in different models. Table 2: relationship between pH and all-cause mortality in different models. Table 3: relationship between lactate and all-cause mortality in different models. Table 4: relationship between urine ketone bodies and all-cause mortality in different models.

## References

[1] McDonald J. S., McDonald R. J., Williamson E. E., Kallmes D. F., Kashani K. (2017). Post-contrast acute kidney injury in intensive care unit patients: a propensity score-adjusted study. *Intensive Care Medicine*.

[2] Odutayo A., Wong C. X., Farkouh M. (2017). AKI and long-term risk for cardiovascular events and mortality. *Journal of the American Society of Nephrology*.

[3] Uchino S., Kellum J. A., Bellomo R. (2005). Acute renal failure in critically ill patients: a multinational, multicenter study. *JAMA*.

[4] Hofhuis J. G. M., van Stel H. F., Schrijvers A. J. P., Rommes J. H., Spronk P. E. (2013). The effect of acute kidney injury on long-term health-related quality of life: a prospective follow-up study. *Critical Care*.

[5] White L. E., Hassoun H. T., Bihorac A. (2013). Acute kidney injury is surprisingly common and a powerful predictor of mortality in surgical sepsis. *The Journal of Trauma and Acute Care Surgery*.

[6] Hu Y., Liu H., Fu S., Wan J., Li X. (2017). Red blood cell distribution width is an independent predictor of AKI and mortality in patients in the coronary care unit. *Kidney & Blood Pressure Research*.

[7] Wu B., Chen J., Yang Y. (2019). Biomarkers of acute kidney injury after cardiac surgery: a narrative review. *BioMed Research International*.

[8] Kraut J. A., Madias N. E. (2007). Serum anion gap: its uses and limitations in clinical medicine. *Clinical Journal of the American Society of Nephrology*.

[9] Salem M. M., Mujais S. K. (1992). Gaps in the anion gap. *Archives of Internal Medicine*.

[10] Farwell W. R., Taylor E. N. (2008). Serum bicarbonate, anion gap and insulin resistance in the National Health and Nutrition Examination Survey. *Diabetic Medicine*.

[11] Abramowitz M. K., Hostetter T. H., Melamed M. L. (2012). Lower serum bicarbonate and a higher anion gap are associated with lower cardiorespiratory fitness in young adults. *Kidney International*.

[12] Park M., Jung S. J., Yoon S., Yun J. M., Yoon H. J. (2015). Association between the markers of metabolic acid load and higher all-cause and cardiovascular mortality in a general population with preserved renal function. *Hypertension Research*.

[13] Mohr N. M., Vakkalanka J. P., Faine B. A. (2018). Serum anion gap predicts lactate poorly, but may be used to identify sepsis patients at risk for death: a cohort study. *Journal of Critical Care*.

[14] Yang S. W., Zhou Y. J., Zhao Y. X. (2017). The serum anion gap is associated with disease severity and all-cause mortality in coronary artery disease. *Journal of Geriatric Cardiology*.

[15] Chen Q., Chen Q., Li L. (2018). Serum anion gap on admission predicts intensive care unit mortality in patients with aortic aneurysm. *Experimental and Therapeutic Medicine*.

[16] Abramowitz M. K., Hostetter T. H., Melamed M. L. (2012). The serum anion gap is altered in early kidney disease and associates with mortality. *Kidney International*.

[17] Wang B., Li D., Gong Y., Ying B., Cheng B. (2019). Association of serum total and ionized calcium with all-cause mortality incritically ill patients with acute kidney injury. *Clinica Chimica Acta*.

[18] Wang B., Gong Y., Ying B., Cheng B. (2019). Relation between red cell distribution width and mortality in critically ill patients with acute respiratory distress syndrome. *BioMed Research International*.

[19] Wang B., Lu H., Gong Y., Ying B., Cheng B. (2018). The association between red blood cell distribution width and mortality in critically ill patients with acute kidney injury. *BioMed Research International*.

[20] Johnson A. E. W., Pollard T. J., Shen L. (2016). MIMIC-III, a freely accessible critical care database. *Scientific Data*.

[21] Allard J., Cotin S., Faure F. (2007). SOFA--an open source framework for medical simulation. *Studies in Health Technology and Informatics*.

[22] Le Gall J. R., Lemeshow S., Saulnier F. (1993). A new simplified acute physiology score (SAPS II) based on a European/North American multicenter study. *JAMA*.

[23] Agoritsas T., Merglen A., Shah N. D., O'Donnell M., Guyatt G. H. (2017). Adjusted analyses in studies addressing therapy and harm: users' guides to the medical literature. *JAMA*.

[24] Banerjee T., Crews D. C., Wesson D. E. (2019). Elevated serum anion gap in adults with moderate chronic kidney disease increases risk for progression to end-stage renal disease. *American Journal of Physiology-Renal Physiology*.

[25] Al-Jaghbeer M., Kellum J. A. (2015). Acid-base disturbances in intensive care patients: etiology, pathophysiology and treatment. *Nephrology, Dialysis, Transplantation*.

[26] Gabow P. A., Kaehny W. D., Fennessey P. V., Goodman S. I., Gross P. A., Schrier R. W. (1980). Diagnostic importance of an increased serum anion gap. *The New England Journal of Medicine*.

[27] Chawla L. S., Bellomo R., Bihorac A. (2017). Acute kidney disease and renal recovery: consensus report of the acute disease quality initiative (ADQI) 16 workgroup. *Nature Reviews Nephrology*.

[28] Sun D. Q., Zhang L., Zheng C. F. (2019). Metabolic acidosis in critically ill cirrhotic patients with acute kidney injury. *Journal of Clinical and Translational Hepatology*.

[29] Levy B., Gibot S., Franck P., Cravoisy A., Bollaert P. E. (2005). Relation between muscle Na^+^K^+^ ATPase activity and raised lactate concentrations in septic shock: a prospective study. *The Lancet*.

[30] Kurtz I., Maher T., Hulter H. N., Schambelan M., Sebastian A. (1983). Effect of diet on plasma acid-base composition in normal humans. *Kidney International*.

[31] Vincent J.-L., Quintairos e Silva A., Couto L., Taccone F. S. (2016). The value of blood lactate kinetics in critically ill patients: a systematic review. *Critical Care*.

[32] Seheult J., Fitzpatrick G., Boran G. (2017). Lactic acidosis: an update. *Clinical Chemistry and Laboratory Medicine*.

