# Supplementary material for: Serum Anion Gap Predicts All-Cause Mortality in Critically Ill Patients with Acute Kidney Injury: Analysis of the MIMIC-III Database
Source: Dis Markers. 2020 Jan 19;2020:6501272. doi: 10.1155/2020/6501272 (PMC6995483; doi:10.1155/2020/6501272)
Supplement: Supplementary Materials — Table 1: relationship between bicarbonate and all-cause mortality in different models. Table 2: relationship between pH and all-cause mortality in different models. Table 3: relationship between lactate and all-cause mortality in different models. Table 4: relationship between urine ketone bodies and all-cause mortality in different models. [file 6501272.f1.pdf]

Table 1 Relationship between bicarbonate and all-cause mortality in different models

Variable	Crude Model		Model I		Model II	
	HR (95%CIs)	*P* value	HR (95%CIs)	*P* value	HR (95%CIs)	*P* value
30-day all-cause mortality						
Bicarbonate (tertile), mmol/L						
<22	1.0(ref)		1.0(ref)		1.0(ref)	
≥22, <26	0.66 (0.60, 0.73)	<0.0001	0.64 (0.58, 0.71)	<0.0001	1.01 (0.90, 1.12)	0.9237
≥26	0.56 (0.51, 0.62)	<0.0001	0.54 (0.49, 0.59)	<0.0001	1.00 (0.89, 1.13)	0.9742
P for trend	<0.0001		<0.0001		0.9680	
90-day all-cause mortality						
Bicarbonate (tertile), mmol/L						
<22	1.0(ref)		1.0(ref)		1.0(ref)	
≥22, <26	0.68 (0.63, 0.74)	<0.0001	0.66 (0.61, 0.72)	<0.0001	1.02 (0.93, 1.12)	0.6847
≥26	0.60 (0.56, 0.66)	<0.0001	0.57 (0.53, 0.62)	<0.0001	1.03 (0.93, 1.13)	0.6138
P for trend	<0.0001		<0.0001		0.6074	
365-day all-cause mortality						
Bicarbonate (tertile), mmol/L						
<22	1.0(ref)		1.0(ref)		1.0(ref)	
≥22, <26	0.72 (0.67, 0.78)	<0.0001	0.70 (0.65, 0.75)	<0.0001	1.03 (0.95, 1.12)	0.5034
≥26	0.69 (0.64, 0.74)	<0.0001	0.65 (0.61, 0.70)	<0.0001	1.08 (0.99, 1.17)	0.0909

P for trend	<0.0001	<0.0001	0.0930

HR: hazard ratio; CI: confidence interval.

Models were derived from Cox proportional hazards regression models.

Crude model adjust for: none.

Adjust I model adjust for: age, gender.

Adjust II model adjust for: age, gender, acute kidney injury stage, congestive heart failure, coronary artery disease, liver disease, stroke, respiratory failure, pneumonia, SIRS, potassium, albumin, platelet, BUN, PT, INR, APTT, WBC, creatinine, bilirubin, SPO2, heart rate, systolic blood pressure, diastolic blood pressure, respiratory rate, temperature, Elixhauser comorbidity index, SOFA, SAPSII.

In Table 1, after adjusting for more relevant confounding factors, we found that bicarbonate levels were not independently associated with 30, 90 and 365-day all-cause mortality in critically ill patients with AKI.

Table 2 Relationship between pH and all-cause mortality in different models

Variable	Crude Model		Model I		Model II	
	HR (95%CIs)	*P* value	HR (95%CIs)	*P* value	HR (95%CIs)	*P* value
30-day all-cause mortality						
pH (tertile)						
<7.32	1.0(ref)		1.0(ref)		1.0(ref)	
≥7.32, <7. 40	1.08 (0.81, 1.44)	0.6181	0.87 (0.65, 1.17)	0.3702	0.85 (0.58, 1.23)	0.3887
≥7.40	1.40 (1.08, 1.81)	0.0107	1.02 (0.78, 1.33)	0.8953	0.96 (0.66, 1.39)	0.8230
P for trend	0.0135		0.9221		0.7431	
90-day all-cause mortality						
pH (tertile)						
<7.32	1.0(ref)		1.0(ref)		1.0(ref)	
≥7.32, <7. 40	1.14 (0.83, 1.58)	0.4209	0.94 (0.68, 1.31)	0.7266	0.93 (0.62, 1.39)	0.7225
≥7.40	1.36 (1.02, 1.82)	0.0387	1.03 (0.76, 1.38)	0.8641	0.92 (0.61, 1.40)	0.6989
P for trend	0.0419		0.8789		0.6855	
365-day all-cause mortality						
pH (tertile)						
<7.32	1.0(ref)		1.0(ref)		1.0(ref)	
≥7.32, <7. 40	1.12 (0.86, 1.45)	0.4115	0.88 (0.68, 1.15)	0.3618	0.86 (0.62, 1.21)	0.4010
≥7.40	1.44 (1.14, 1.82)	0.0025	1.01 (0.79, 1.28)	0.9561	0.92 (0.65, 1.29)	0.6179

P for trend	0.0033	0.9786	0.5614

HR: hazard ratio; CI: confidence interval.

Models were derived from Cox proportional hazards regression models.

Crude model adjust for: none.

Adjust I model adjust for: age, gender.

Adjust II model adjust for: age, gender, acute kidney injury stage, congestive heart failure, coronary artery disease, liver disease, stroke, respiratory failure, albumin, platelet, BUN, PT, INR, APTT, WBC, creatinine, bicarbonate, systolic blood pressure, diastolic blood pressure, respiratory rate, temperature, Elixhauser comorbidity index, SOFA, SAPSII.

In Table 2, after adjusting for more relevant confounding factors, we found that pH values were not independently associated with 30, 90 and 365-day all-cause mortality in critically ill patients with AKI.

Table 3 Relationship between lactate and all-cause mortality in different models

Variable	Crude Model		Model I		Model II	
	HR (95%CIs)	*P* value	HR (95%CIs)	*P* value	HR (95%CIs)	*P* value
30-day all-cause mortality						
Lactate (tertile), mmol/L						
<1.9	1.0(ref)		1.0(ref)		1.0(ref)	
≥1.9, <3.5	1.44 (1.28, 1.62)	<0.0001	1.43 (1.27, 1.61)	<0.0001	1.26 (1.10, 1.44)	0.0006
≥3.5	1.98 (1.77, 2.22)	<0.0001	2.02 (1.81, 2.27)	<0.0001	1.26 (1.10, 1.44)	0.0009
P for trend	<0.0001		<0.0001		0.0153	
90-day all-cause mortality						
Lactate (tertile), mmol/L						
<1.9	1.0(ref)		1.0(ref)		1.0(ref)	
≥1.9, <3.5	1.37 (1.24, 1.51)	<0.0001	1.36 (1.23, 1.50)	<0.0001	1.21 (1.08, 1.35)	0.0008
≥3.5	1.72 (1.56, 1.89)	<0.0001	1.76 (1.60, 1.94)	<0.0001	1.17 (1.04, 1.31)	0.0089
P for trend	<0.0001		<0.0001		0.0903	
365-day all-cause mortality						
Lactate (tertile), mmol/L						
<1.9	1.0(ref)		1.0(ref)		1.0(ref)	
≥1.9, <3.5	1.23 (1.14, 1.34)	<0.0001	1.22 (1.12, 1.33)	<0.0001	1.16 (1.06, 1.27)	0.0015
≥3.5	1.42 (1.31, 1.54)	<0.0001	1.45 (1.34, 1.58)	<0.0001	1.08 (0.97, 1.19)	0.1474

P for trend	<0.0001	<0.0001	0.6246

HR: hazard ratio; CI: confidence interval.

Models were derived from Cox proportional hazards regression models.

Crude model adjust for: none.

Adjust I model adjust for: age, gender.

Adjust II model adjust for: age, gender, acute kidney injury stage, congestive heart failure, coronary artery disease, liver disease, stroke, respiratory failure, pneumonia, SIRS, potassium, albumin, platelet, bicarbonate, BUN, PT, INR, APTT, WBC, creatinine, bilirubin, SPO2, heart rate, systolic blood pressure, diastolic blood pressure, respiratory rate, temperature, renal replacement therapy, Elixhauser comorbidity index, SOFA, SAPSII.

In Table 3, after adjusting for more relevant confounding factors, we found a positive correlation between lactate levels and 30, 90 day all-cause mortality in critically ill patients with AKI.

Table 4 Relationship between urine ketone bodies and all-cause mortality in different models

Variable	Crude Model		Model I		Model II	
	HR (95%CIs)	*P* value	HR (95%CIs)	*P* value	HR (95%CIs)	*P* value
30-day all-cause mortality						
Urine ketone bodies (tertile), mg/dL						
<15	1.0(ref)		1.0(ref)		1.0(ref)	
≥15, <50	1.96 (1.50, 2.55)	<0.0001	1.58 (1.21, 2.06)	0.0008	1.25 (0.88, 1.79)	0.2068
≥50	1.98 (1.41, 2.77)	<0.0001	1.48 (1.05, 2.09)	0.0234	1.36 (0.88, 2.09)	0.1670
P for trend	<0.0001		0.0009		0.1502	
90-day all-cause mortality						
Urine ketone bodies (tertile), mg/dL						
<15	1.0(ref)		1.0(ref)		1.0(ref)	
≥15, <50	1.88 (1.40, 2.52)	<0.0001	1.54 (1.15, 2.07)	0.0041	1.18 (0.80, 1.73)	0.4032
≥50	1.78 (1.22, 2.61)	0.0031	1.38 (0.94, 2.03)	0.1012	1.36 (0.85, 2.18)	0.2009
P for trend	<0.0001		0.0060		0.2727	
365-day all-cause mortality						
Urine ketone bodies (tertile), mg/dL						
<15	1.0(ref)		1.0(ref)		1.0(ref)	
≥15, <50	1.80 (1.43, 2.27)	<0.0001	1.44 (1.13, 1.82)	0.0026	1.17 (0.85, 1.60)	0.3370
≥50	1.86 (1.38, 2.51)	<0.0001	1.37 (1.01, 1.86)	0.0413	1.20 (0.81, 1.78)	0.3650

P for trend	<0.0001	0.0027	0.2962

HR: hazard ratio; CI: confidence interval.

Models were derived from Cox proportional hazards regression models.

Crude model adjust for: none.

Adjust I model adjust for: age, gender.

Adjust II model adjust for: age, gender, acute kidney injury stage, congestive heart failure, coronary artery disease, liver disease, stroke, respiratory failure, albumin, bicarbonate, creatinine, systolic blood pressure, diastolic blood pressure, respiratory rate, SOFA, SAPSII.

In Table 4, after adjusting for more relevant confounding factors, we found that urine ketone bodies levels were not independently associated with 30, 90 and 365-day all-cause mortality in critically ill patients with AKI.
